# Pocket CLARITY enables distortion-mitigated cardiac microstructural tissue characterization of large-scale specimens

**DOI:** 10.3389/fcvm.2022.1037500

**Published:** 2022-11-14

**Authors:** Joan J. H. Kim, Shestruma Parajuli, Aman Sinha, Mohammed Mahamdeh, Maaike van den Boomen, Jaume Coll-Font, Lily Shi Chen, Yiling Fan, Robert A. Eder, Kellie Phipps, Shiaulou Yuan, Christopher Nguyen

**Affiliations:** ^1^Cardiovascular Research Center, Massachusetts General Hospital, Charlestown, MA, United States; ^2^A.A. Martinos Center for Biomedical Imaging, Massachusetts General Hospital, Boston, MA, United States; ^3^Department of Radiology, University Medical Center Groningen, University of Groningen, Groningen, Netherlands; ^4^Department of Medicine, Harvard Medical School, Boston, MA, United States; ^5^Department of Mechanical Engineering, Massachusetts Institute of Technology, Cambridge, MA, United States; ^6^Division of Health Science Technology, Harvard-Massachusetts Institute of Technology, Cambridge, MA, United States; ^7^Cardiovascular Innovation Research Center, Heart, Vascular, and Thoracic Institute, Cleveland Clinic, Cleveland, OH, United States

**Keywords:** CLARITY, helicity, microstructure, distortion, lightsheet microscopy

## Abstract

Molecular phenotyping by imaging of intact tissues has been used to reveal 3D molecular and structural coherence in tissue samples using tissue clearing techniques. However, clearing and imaging of cardiac tissue remains challenging for large-scale (>100 mm^3^) specimens due to sample distortion. Thus, directly assessing tissue microstructural geometric properties confounded by distortion such as cardiac helicity has been limited. To combat sample distortion, we developed a passive CLARITY technique (Pocket CLARITY) that utilizes a permeable cotton mesh pocket to encapsulate the sample to clear large-scale cardiac swine samples with minimal tissue deformation and protein loss. Combined with light sheet auto-fluorescent and scattering microscopy, Pocket CLARITY enabled the characterization of myocardial microstructural helicity of cardiac tissue from control, heart failure, and myocardial infarction in swine. Pocket CLARITY revealed with high fidelity that transmural microstructural helicity of the heart is significantly depressed in cardiovascular disease (CVD), thereby revealing new insights at the tissue level associated with impaired cardiac function.

## Introduction

Cardiovascular disease (CVD) is the leading cause of death globally (1). Clinically, the impact of CVD is assessed by measuring the pumping efficiency of the heart using ejection fraction (a volumetric ratio of the stroke volume to the end-diastolic volume (2). Ejection fraction measurements provide valuable insights about the functional status of the heart, but yield minimal information about microstructural changes and/or tissue regeneration after an infarct which is a valuable aid in detection and diagnosis. Instead, microstructural changes are commonly studied using histology where the tissue is rendered into a series of thin two-dimensional slices (2–25 μm thick) that are stained and observed under a microscope (3). The drawback of studying 2D sections is the absence of 3D structural information that is needed to better understand disease progression (4). In addition, the low sampling nature of examining sparse 2D sections increases the chances of missing or misinterpreting disease queues. Earlier efforts to perform 3D histology utilized microscopy techniques like knife-edge scanning microscopy and confocal microscopy among others (5). However, the relatively slow acquisition rate, destructive nature and low optical penetration depth of these techniques has hindered wide scale adoption. Further, light absorption and scattering are additional limiting factors for imaging of samples larger than 100–200 μm. This is due to refractive index mismatches between the sample and the imaging media as well as refractive index changes within the sample itself due to its inhomogeneous molecular composition that includes lipids, proteins and other components. One solution to reduce light absorption and scattering is to homogenize the samples to render them transparent, a process known as tissue clearing. Although tissue clearing dates back to early 1900s, it recently gained momentum due to advances in microscopy and the growing interest in volumetric imaging. Novel tissue clearing approaches combined with advanced imaging techniques allows for non-destructive 3D histological analysis of tissue and whole organs and is routinely performed using a spectrum of clearing techniques such as CLARITY (6–8), BABB (9), CUBIC (10), and 3DISCO (11). These techniques have been applied to several organs across different species, including the brain (6), the nervous system (6), the liver and other organs (12) of mice and mini-pigs (13). In a recent work, whole human organs were also cleared using the SHANEL clearing method (14) as well as human embryos (15). Also, the SHIELD clearing protocol has been used to analyze the synoptic architecture of mice brain (16) as well as to analyze mice hearts. While the method does provide transparency with minimized distortions, there are still some limitations regarding the homogenous staining (17). Critically, many of these clearing methods have been coupled with lightsheet microscopy, a fluorescence imaging technique with high-speed optical sectioning capability, to enable rapid volumetric imaging of whole organs and embryos in TOTO. These applications have enabled optical 3D histology and molecular phenotyping to determine the 3D molecular and structural coherence in organs and tissues.

The degree of clearing ultimately impacts image quality and depends on many factors such as the clearing solution, tissue type, and size of tissue. Dense large tissue, such as the intact heart, require additional clearing time and is more prone to geometric distortion of the original sample. Distortion is a common side effect of clearing procedures usually in the form of expansion or shrinkage. For example, solvent based techniques such as BABB and iDISCO often cause sample shrinkage whereas hydrogel embedding techniques like CLARITY lead to sample expansion (17). Similarly, other tissue clearing techniques such as CUBIC and ClearT are known to cause tissue swelling and tissue volume changes, respectively (18). In cleared specimens, tissue distortion occurs in a non-isotropic fashion and is more detrimental as the sample size increases. Distortion can especially have a significant impact when quantifying microstructural organization where geometrical integrity of the sample is required to characterize the underlying tissue architecture such as cardiomyocyte orientation in the heart. This applies to hearts in particular since, compared to other organs, cardiac tissue clearing is more challenging due to its dense composition and thick tissue structure (13). For small mammalian models, like mice and rats (6), tissue distortion has a minor impact on sample shape. However, for larger mammalian models such as swine, which is a pivotal animal model for clinical research on heart conditions, distortion is a major hurdle for cardiac tissue analysis *via* tissue clearing. Successful clearing of swine hearts has been previously demonstrated, enabling 3D assessment of the vascular densities and diameters in healthy and infarcted myocardium (19). Despite this, severe tissue distortions were observed on the cleared sections (19). While these distortions had minor effects on vascular analysis, they impact the quantification of microstructural organization of myocardial fibers throughout the transmural layers and are likely to result in systemic errors (12). Even though many alternative techniques were introduced to mitigate tissue distortion (20), a protocol to preserve macrostructure of cardiac tissue and internal microstructure that supports immunostaining and maintains fluorescence protein signal has not yet been established (21, 22).

**FIGURE 1:**
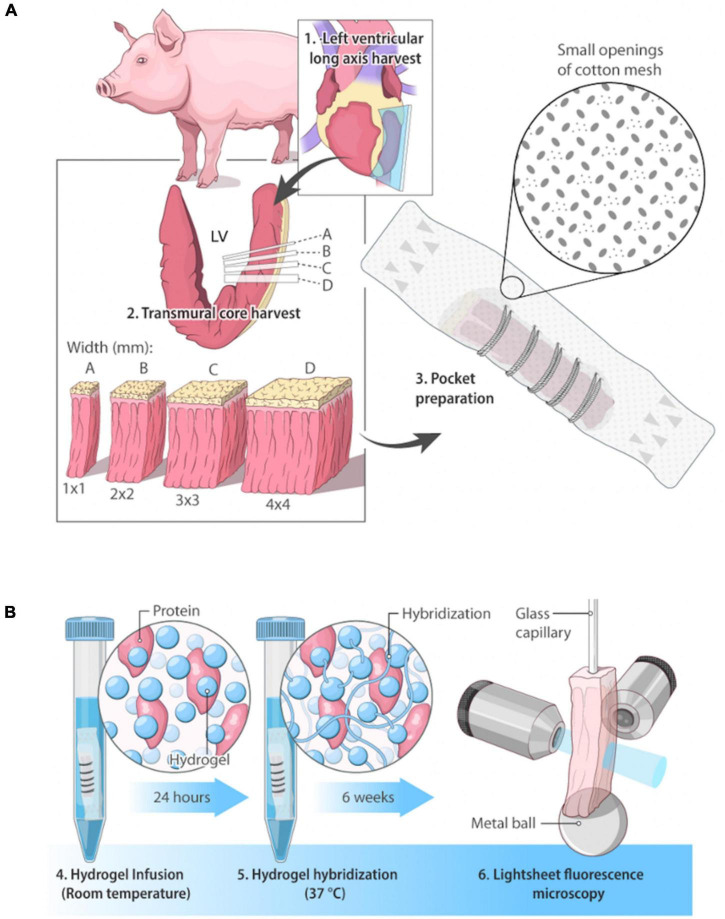
Overview of swine cardiac tissue clearing and imaging. **(A)** Pocket CLARITY Tissue Preparation. Cardiac tissue of a Yucatan swine on the lateral side was harvested in sections of widths of 1 mm × 1 mm, 2 mm × 2 mm, 3 mm × 3 mm, and 4 mm × 4 mm. Lengths of tissue samples were the kept at the lengths of the heart wall, representing its full thickness. Samples were hand-stitched into cotton mesh (thus creating the “pocket”) to prevent tissue distortion during the lipid clearing process. **(B)** Pocket CLARITY Tissue Clearing Process and Imaging. Samples in their pockets were placed in hydrogel monomer for 24 h at room temperature to allow for hydrogel infusion, then switched to fresh hydrogel solution for 3 h at 37°C to allow for hydrogel hybridization. Samples were then “washed” with an 8% detergent sodium dodecyl sulfate (SDS) clearing solution to begin lipid removal. Samples were kept at 37°C for the entirety of the 6-week lipid removal process. The clearing solution was changed every 24 h for the next 48 h to ensure removal of PFA, and then changed every 3–4 days for optimal lipid removal. After clearing, samples were removed from pockets, mounted on a glass capillary and imaged using lightsheet fluorescence microscopy.

As distortion-free whole swine heart clearing for structural studies remains challenging, an alternative approach is to harvest thin transmural cardiac core samples that are more amenable for clearing. Despite allowing for improved clearing quality (17), transmural samples also suffer from distortion for sizes larger than 100 mm (3) albeit to a lesser degree compared to whole hearts. Here we introduce an easy-to-implement and cost-efficient passive clearing method based on a conventional CLARITY protocol that minimizes sample distortion at large-scales (> 100 mm^3^), which we coined “Pocket CLARITY.” CLARITY is among the most used clearing techniques since it: (1) can be applied to various tissue types including hearts, (2) preserves expressed fluorescent proteins and (3) allows for immunofluorescence staining. With Pocket CLARITY, the structure of the cleared sample is maintained by “pocketing” the sample into a permeable cotton mesh that limits tissue distortion by mechanically restraining the sample to its original shape and allowing for a controlled expansion along a direction inconsequential to myocardial microstructural helical characterization (Figure 1). We demonstrate the performance and advantages of the Pocket CLARITY on swine transmural myocardium samples in comparison to conventional CLARITY by measuring transmural myocardial microstructure organization throughout a range of samples sizes for both healthy and diseased tissue *via* lightsheet microscopy.

## Results

### CLARITY distorts large-scale specimens reducing image quality and quantification

**FIGURE 2:**
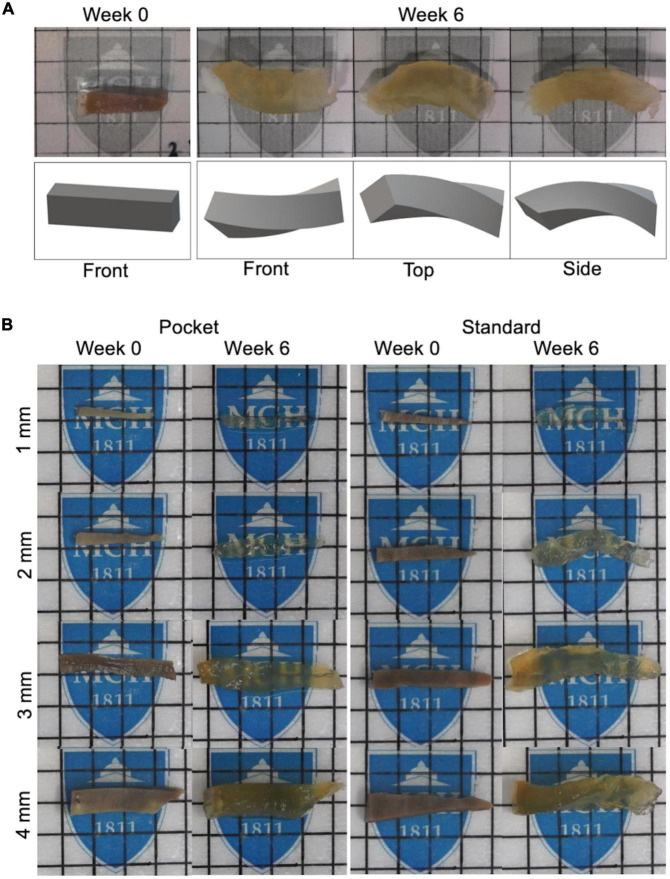
Clearing protocol of pocket CLARITY and CLARITY and resulting tissue distortion after 6 weeks. **(A)** Demonstration of Tissue Distortion Caused by Standard CLARITY. Images and 3D models of the geometric distortion exhibited by a 3 mm left ventricle sample having undergone CLARITY (32). **(B)** CLARITY and Pocket CLARITY Pre and Post Clearing. (Left panel) Pocket CLARITY of transmural swine left ventricle core at Week 0 and Week 6 of clearing. Images were taken before samples were stitched in, and after removal from the pocket. Each lattice indicates 1 mm × 1 mm. (Right panel) CLARITY of transmural swine left ventricle core at Week 0 and Week 6 of clearing. Each lattice scale is 5 mm × 5 mm. For all images, specimens are oriented with epicardium to the left and endocardium to the right.

As a first step toward devising an approach for minimizing tissue distortion, we performed clearing on porcine transmural (endocardium to epicardium) heart samples varying in size from 1 to 4 mm cross section widths and lengths of up to 20 mm using a standard CLARITY protocol over the course of 6 weeks (Figure 2). After 6 weeks of clearing, CLARITY cleared samples were scanned with an in-house built lightsheet microscope in the longitudinal direction acquiring an entire z-stack before tiling across the entire length of each sample. Then, the acquired images were virtually resampled cross-sectionally to reveal thousands of transmural layers from the endocardium to epicardium. The thousands of cross-sectional images were then used to calculate the helix angle (HA) at every transmural depth using a 2D structure tensor analysis to calculate the mean fiber orientation for each image. HA is a critically important measure in characterizing the heart’s microstructural architecture relies heavily on accurate estimation of the myocardial fiber orientation at each transmural depth and thus, is highly susceptible to tissue distortion. Only the 1mm cross sectional conventionally CLARITY cleared samples revealed cardiac fiber structures throughout the transmural layers but had superimposed streaking-like artifacts that disrupted the automated aforementioned HA quantification (Supplementary Figure 2). Furthermore, standard CLARITY cleared specimens exhibited severe distortions for all cross-sectional widths between Week 1 and 3 of clearing (Supplementary Figure 1) and severe distortion after 6 weeks of clearing (Figure 2A). While the 1 mm standard CLARITY samples were able to yield images, the associated distortions prevented any meaningful analyses (Supplementary Figure 2).

### Pocket CLARITY reduces distortion from clearing

**FIGURE 3:**
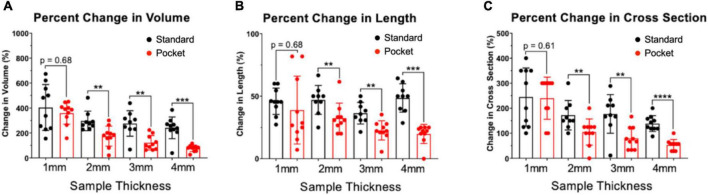
Percent changes in volume, length, and cross-section of CLARITY and pocket CLARITY samples. **(A)** Percent Change in Volume. After 6 weeks of passive clearing, CLARITY 2, 3, and 4 mm samples (*n* = 78) exhibited significantly increased percent change in volume (296 ± 78, 275 ± 102, and 241 ± 87, respectively) compared with pocket CLARITY samples (174 ± 80, 121 ± 58, and 76 ± 26, respectively). For 1 mm standard and pocket CLARITY samples, clearing resulted in no significance percent change in volume (404 ± 184 vs. 359 ± 90). **(B)** Percent Change in Length. Percent change in length of sample sizes from CLARITY (*n* = 38) and Pocket CLARITY (*n* = 40) between Week 0 and Week 6. For the 2 mm, 3 mm, 4 mm samples, standard cleared samples showed an increase percent change in length (47 ± 12, 36 ± 9, and 49 ± 11) compared to the pocket cleared samples (33 ± 12, 25 ± 5, and 22 ± 3). No significant difference in percent change in length for the 1 mm samples between standard cleared (45.93 ± 10.52) and pocket cleared samples (36.15 ± 27.23). Measurements of sample length were taken from the longest points of sample growth. **(C)** Percent Change of Cross-Section. Percent change of tissue width-height cross section from CLARITY (*n* = 38) and Pocket CLARITY (*n* = 40) between Week 0 and Week 6 of clearing. For the 2, 3, and 4 mm samples, standard cleared samples showed an increase percent change in cross-section (172 ± 59, 177 ± 77, and 138 ± 32) compared to the pocket cleared samples (105 ± 52, 80 ± 44, and 54 ± 22), while there was no significant difference in percent change in cross-section for the 1 mm standard (245 ± 116) and pocket samples (240 ± 84). Measurements were taken from the tallest and widest points of sample growth. **significant at *p* < 0.01, ***significant at *p* < 0.001, ****significant at *p* < 0.0001.

Pocket CLARITY improved tissue clearing (Figure 2) with significantly less distortion for larger samples (Figure 3) compared to CLARITY, resulting in the ability to image the various tissues. For 1 mm samples, Pocket CLARITY allowed for clearing with no significant difference in distortion represented by changes in volume, length, and cross section, while the 2 mm samples exhibited similar clearing but significantly decreased distortion (decrease of 56, 30, and 39% for volume, length and cross section respectively). For 3 and 4 mm samples, partial clearing was observed but significantly decreased distortion (relative decrease 41, 56, and 68% for the 3 mm and relative decrease of 68, 55, and 61% for the 4 mm samples). Furthermore, Pocket CLARITY tissue samples maintained their overall shape compared with CLARITY (Figures 2B, 3).

**FIGURE 4:**
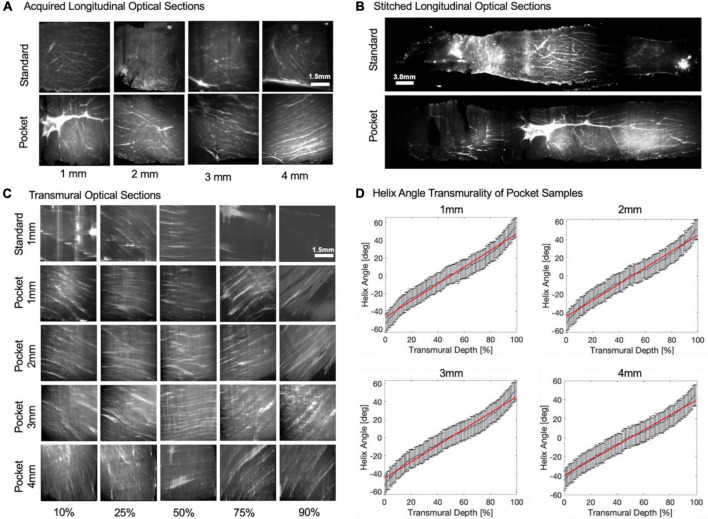
Longitudinal/transmural imaging and helix angle maps of cardiac tissues. **(A)** Raw longitudinal optical sections. Raw-lightsheet microscopy longitudinal optical sections of 1, 2, 3, and 4 mm Standard and Pocket cleared samples before stitching. **(B)** Stitched longitudinal optical sections. Longitudinal optical sections were stitched from epicardium to endocardium for Standard and Pocket cleared samples. Representative 1 mm sample is shown. **(C)** Transmural optical sections. Longitudinal optical sections were then resampled orthogonally into cross sections to reveal transmural optical sections of Standard and Pocket cleared samples. Representative 1 mm Standard cleared sample is shown compared with 1, 2, 3, and 4 mm Pocket cleared samples. Transmural changes in the fiber orientation are observed reflecting the change from left-handed helix to right-handed helix. Standard cleared 2, 3, and 4 mm samples result in poor image quality obfuscating helical fiber orientations (see Supplementary Figure 1) **(D)** Helix Angle Transmurality of Pocket Samples. Quantification of the helix angle from epicardium to endocardium for representative 1, 2, 3, and 4 mm Pocket cleared samples. Red line indicates the helix angle transmurality calculated as the slope.

By maintaining the sample shape, Pocket CLARITY allowed for cardiac fiber microstructures to be visualized with Lightsheet Fluorescence Microscopy (LSFM) for all sample sizes (1–4 mm) (Figure 4) across the continuum of the sample from endocardium to epicardium layers of the transmural core. Stitching and reformatting of the samples allowed visualization of cardiac fiber microstructures at thousands of transmural optical sections from epicardium to endocardium. Furthermore, quantifying the primary eigenvector of the structure tensor of each optical section revealed a continual change of HA from left handedness to right handedness for all sample sizes ranging from (–51°± 13 to 53°± 11). The slope of the HA values from endocardium to epicardial layers or the helix angle transmurality (HAT) were similar across 1, 2, 3, and 4 mm samples (0.90 ± 0.07°/%, 0.94°± 0.07°/%, 0.91 ± 0.03°/%, and 0.89 ± 0.06°/% degrees of helix angle per percent of tissue from epicardium to endocardium). The HAT values were not statistically significant when compared within the various sample sizes.

### Pocket CLARITY has no impact on protein loss

Total percent protein loss was measured for both CLARITY and Pocket CLARITY samples throughout the 6-week clearing process. Chung et al. reported 8% decrease in protein content and indicated that chemical tethering of biomolecules into hydrogel mesh can enhance the preservation of molecular tissue components. It was unclear if pocket CLARITY could interfere with the hydrogel bonding, and we therefore performed protein loss analysis on both CLARITY and pocket CLARITY samples. Protein loss for pocket CLARITY was found to be 28.718.3, 15.7, and 20.9% for 1, 2, 3, and 4 mm samples, respectively, when normalized per volume for the 6-week clearing period. These values were not statistically significant when compared between the four sizes. The *p*-value was found to be 0.4 for 1 mm vs. 2 mm, 0.3 for 1 mm vs. 3 mm, 0.8 for 1 mm vs. 4 mm, 0.7 for 2 mm vs. 3 mm, 0.9 for 2 mm vs. 4 mm, 0.8 for 3 mm vs. 4 mm (Table 1).

**TABLE 1:** Pocket CLARITY and CLARITY protein loss.

	Size	Percent loss	Size comparison	*P*-value
Pocket CLARITY	1 mm	28.7%	1 vs. 2	0.4
	2 mm	18.3%	1 vs. 3	0.3
			1 vs. 4	0.8
	3 mm	15.7%	2 vs. 3	0.7
	4 mm	20.9%	2 vs. 4	0.9
			3 vs. 4	0.8
CLARITY	1 mm	18.6%	1 vs. 2	0.8
	2 mm	16.2%	1 vs. 3	0.1
			1 vs. 4	0.1
	3 mm	28.7%	2 vs. 3	0.2
	4 mm	31.5%	2 vs. 4	0.2
			3 vs. 4	0.2

The percent protein loss for standard CLARITY was found to be 18.6, 16.2, 28.7, and 31.5 for 1, 2, 3, and 4 mm samples, respectively. These values were also not statistically significant when compared between the four size groups. The *p*-value was found to be 0.8 for 1 mm vs. 2 mm, 0.1 for 1 mm vs. 3 mm, 0.1 for 1 mm vs. 4 mm, 0.2 for 2 mm vs. 3 mm, 0.2 for 2–4 mm, 0.2 for 3 mm vs. 4 mm (Table 1).

**FIGURE 5:**
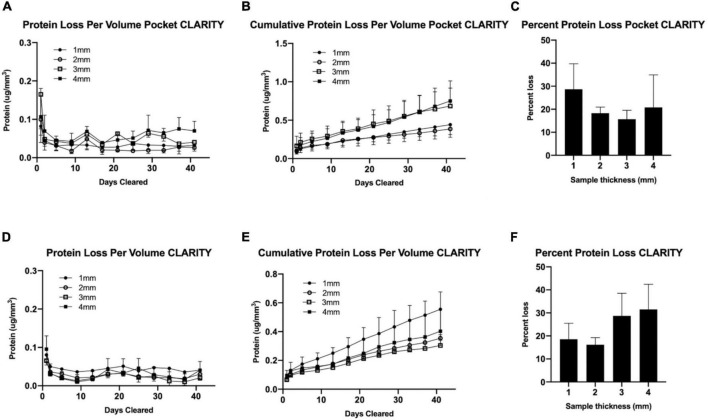
Protein analysis for CLARITY vs. pocket CLARITY. After 6 weeks of passive clearing, SDS samples from Pocket CLARITY (*n* = 12) and CLARITY (*n* = 12) were analyzed to measure Protein loss per volume, Cumulative protein loss per volume and Total Percent protein loss. **(A)** Protein Loss Per Volume Pocket CLARITY. The amount of protein loss for 1, 2, 3, and 4 mm Pocket CLARITY samples over 40 days. **(B)** Cumulative Protein Loss Per Volume Pocket CLARITY. The cumulative amount of protein loss for 1, 2, 3, and 4 mm Pocket CLARITY samples over 40 days. **(C)** Percent Protein Loss Pocket CLARITY. The percent protein loss for the 1, 2, 3, and 4 mm Pocket CLARITY samples. **(D)** Protein Loss Per Volume CLARITY. The amount of protein loss for 1, 2, 3, and 4 mm CLARITY samples over 40 days. **(E)** Cumulative Protein Loss Per Volume CLARITY. The cumulative amount of protein loss for 1, 2, 3, and 4 mm CLARITY samples over 40 days. **(F)** Percent Protein Loss CLARITY. The percent protein loss for the 1, 2, 3, and 4 mm samples after CLARITY. The amount of protein at each time point was determined using the sample plate reader at a wavelength of 562 nm using protocol from the Thermo Scientific™ Pierce™ BCA Protein Assay Kit for **(A)** Pocket CLARITY and **(D)** CLARITY to analyze the protein loss per volume. The protein loss per volume (μg/mm3) was then added at each time point to calculate the Cumulative protein loss per volume for **(B)** CLARITY and **(E)** Pocket CLARITY. The protein loss did not plateau at Week 6 and there was no significant protein loss between 1, 2, 3, and 4 mm at any time point from Week 0 to 6. The total percent of protein loss was then measured for **(C)** Pocket CLARITY and **(F)** CLARITY by extracting the protein from an uncleared sample to determine the original protein content which was then compared to the amount of protein loss in the SDS. The percent protein loss was not statistically significant for CLARITY and Pocket CLARITY when analyzed.

Furthermore, protein loss was compared between CLARITY and pocket CLARITY; the percent losses were not statistically significant between the two methods. The *p*-values were 0.2, 0.4, 0.1, and 0.4 for 1, 2, 3, and 4 mm samples, respectively (Figure 5).

### Pocket CLARITY shows loss of helicity in diseased cardiac tissues

**FIGURE 6:**
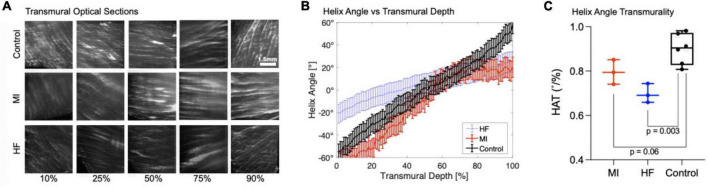
Transmural optical sections and helix angle characterizations of myocardial infarcted and heart failure cardiac samples. **(A)** Transmural optical sections of 1 mm Control, MI, and HF cardiac samples at 10, 25, 50, 75, and 90% of the tissue. The fiber orientation of the control is more pronounced and shows the existence of laminar helical structures positioned in a left-handed (epicardium) to right-handed (endocardium) orientation. The MI samples are similar to the control for 10 and 25%, however the fiber orientation is drastically different toward the endocardium where it does not position in a right-handed orientation. For Heart Failure samples, the fiber orientation is less distinct and have different Helix angle for 10, 25, and 90%. **(B)** Quantification of Helix Angle of Cardiac Fibers. The Helix angles were measured along 10, 25, 50, 75, and 90% of heart Failure, myocardial infarcted, and 1 mm control tissues. **(C)** Helix Angle Transmurality. HAT values for myocardial infarcted, heart failure, and 1 mm control samples.

Pocket CLARITY was used to assess the helicity of control tissue compared to myocardial infarcted (MI), and heart failure (HF) cardiac biopsies from porcine models by quantifying the HAT (Figure 6). Across the entire transmural layers, HF biopsies led to an overall significant decrease in magnitude of HA and HAT (0.70 ± 0.04°/%, *p* < 0.003) compared with control (0.90 ± 0.07°/%). Losses of helicity were found for both MI (0.80 ± 0.06°/%, *p* = 0.06) and HF compared with control (0.90 ± 0.07°/%). Additional information on the HAT values for the different samples can be found in Supplementary Figure 3.

## Discussion

Although the large-scale transmural core samples from both Pocket and standard CLARITY exhibited comparable clearing transparency, the standard CLARITY samples resulted in significant distortion compared to Pocket CLARITY. Although previous studies (6) have shown that CLARITY-induced tissue expansion does not cause non-isotropic distortions, our results indicate that the CLARITY sample distortion severely impacted image quality by introducing artifacts and optical aberrations disrupting any further image analysis to characterize the heart’s helical microstructure. Clearing large-scale samples with Pocket CLARITY significantly reduced sample distortion and improved imaging by restricting sample expansion cross sectionally and by allowing for the sample to expand lengthwise while maintaining the original shape of the sample before clearing. For assessing helical transmural microstructural orientation in cardiac tissue, this lengthwise expansion was acceptable along the transmural direction (i.e., endocardium to epicardium) since quantification of cardiac helicity is normalized to the overall length of the tissue sample.

Cardiac helicity of large-scale swine tissue with densely packed fibers was characterized using Pocket CLARITY by quantifying helix angle at thousands of transmural depths from endocardium to epicardium. This level of depth has not been achieved for swine tissue and confirmed previous findings that loss of microstructural helicity underpins CVD (23, 24). Further studies are needed to fully elucidate the mechanism behind this loss helicity, which potentially could be visualized with Pocket CLARITY combined with immunohistochemistry of key molecular markers. Pocket CLARITY is expected to produce more uniform labeling as it reduces the presence of the highly dense areas generated by the twists and bends from distortions from CLARITY.

Though we demonstrated Pocket CLARITY allows for clearing of large-scale samples of the heart, we have not shown its ability to clear and characterize other tissue types. However, given how tightly packed cardiac tissue is compared to other organs such as the brain, the potential of Pocket CLARITY to efficiently clear other tissue types and provide similar improvements in mitigating distortion is likely. Although there were no deformities visible to the eye, there is a possibility of damage to the tissue surface caused by the cotton pocket and the sewing thread, which could have decreased the overall clearing efficacy. Furthermore, our study only tested at max a cross-sectional width of 4 mm and length of up to 20 mm representing approximately 320 mm (3) of volume. While this is considered a large-scale specimen, it is far from the scale of a whole intact organ from a large animal. However, we speculate using the proposed Pocket approach will be useful for whole organs to potentially control and restrict unwanted expansion, which is conducive for faithful structural tissue analysis.

Pocket CLARITY is a simple technique that dramatically reduces geometric distortion by 50–70% compared to standard CLARITY thereby significantly improving image quality and enabling robust microstructural helical characterization of large-scale cardiac samples from pigs. Furthermore, it allowed novel tissue-level insights into the microstructural helical underpinning of two key types of CVD s by revealing an overall depression of the helicity of the heart. While further validation of Pocket CLARITY technique is required, it is well poised to deliver high throughput imaging of thousands of layers of the myocardium at similar scale to human heart tissue samples. Future directions will include validating Pocket CLARITY’s efficacy in providing exogenous staining as well as in large-scale whole intact organs.

## Materials and methods

### Animals

This study was performed in accordance with institutional and ARRIVE guidelines, and it was approved by the ethical review board of the Massachusetts General Hospital (2009N000238) in 25–30 kg female Yucatan pig. Healthy (*n* = 5), myocardial infarcted (*n* = 4), and heart failure (*n* = 5) swine cardiac tissue samples were obtained in adherence to institutional guidelines. For tissue harvest of each cohort, animals were first anesthetized using Xylazine (2.2 mg/kg), Telazol (4.4 mg/kg) and Atropine (0.04 mg/kg) intramuscularly, followed by continuously inhalation of Isoflurane (1–3%) and euthanasia using pentobarbital euthanasia solution (Euthasol 100 mg/kg IV). Cardiac tissue was obtained immediately upon euthanasia and placed in formaldehyde.

### Myocardial infarction and heart failure induction

For myocardial infarction (MI) induction, a guide catheter was inserted through the introducer and advanced into the ostium of the main left coronary artery. Coronary angiography was performed to assess coronary artery size. Then, an appropriately sized balloon angioplastic catheter advanced over the guidewire through the guide catheter and positioned into the left anterior descending (LAD) coronary artery just distal to the first diagonal branch. Coronary angiogram was performed to confirm angioplastic balloon position. MI creation was then initiated by inflating the balloon to the appropriate size to deprive blood flow distal to the balloon for 80 min at normal physiological body temperature. Total LAD occlusion was confirmed *via* coronary angiogram through the guide catheter approximately every 20 min throughout the occlusion period. Following the 80-min occlusion period, the balloon was deflated to re-establish blood flow to the LAD and induce reperfusion injury to the designated area.

For heart failure induction, a permanent electronic pacemaker was implanted in Yucatan swine by introducing a lead into the right atrium using fluoroscopic guidance. The pacemaker was set to a tachycardia heart rate determined by the animal’s baseline heart rate. Two weeks after the pacemaker was turned on, heart failure was validated by imaging the animal’s heart by MRI.

### CLARITY protocol

Tissue samples were harvested from PFA-fixed heart tissue and cut with a height and width of 1, 2, 3, or 4 mm. Sample lengths were determined by the length from epicardium to endocardium. The clearing of the heart tissue samples followed the third edition of the Hunter Medical Research Institute’s 3D Tissue Clearing with Passive CLARITY handbook (25), based on protocols by Tomer et al. (26) and Yang et al. (27). The samples were placed in 15 mL falcon tubes containing 10 mL hydrogel and left to infuse at room temperature for 24 h to embed the hydrogel monomer. After the initial embedding, tissue samples were placed in fresh hydrogel and flooded with nitrogen gas to eliminate oxygen during polymerization. The samples were then placed in an incubator of 37°C with a rotating drum to provide gentle shaking for 3 h before being removed from the hydrogel and placed in an 8% detergent sodium dodecyl sulfate (SDS) clearing solution to begin lipid removal. Samples were returned to the 37°C incubator with the rotating drum. The clearing solution was changed every 24 h for the next 48 h to ensure removal of PFA, and then changed every 3–4 days for optimal lipid removal (Figure 1B). Before starting the tissue clearing process and after 6 weeks of clearing, the samples were measured to determine their longest points of length, width, and height (Figure 3).

### Pocket CLARITY protocol

Tissue samples of the four different sample sizes described above were harvested from the same heart but an alternative pocket clearing protocol was applied to mitigate tissue deformation during the clearing process. Prior to starting the hydrogel polymerization step, the samples were placed inside a porous fabric (Generic Cotton Mesh, Disposable, SGTA, Item Model Number: 8541897096) which provided a “pocket.” The sample was sewn using a Size 12 needle in the cotton mesh, securing it in place. The samples were placed along their length on the bottom of the cotton mesh and were sewn in to prevent deformation in the height and width, while leaving lengthwise growth unrestricted (Figure 1A).

### Percent changes in volume, length, and cross-section of CLARITY and pocket CLARITY samples

Percent changes in volume, length, and cross-section of CLARITY and Pocket CLARITY samples were calculated using measurements of the height, width, and length of the pre-cleared and cleared samples post 6-weeks of clearing. Measurements were taken of the tallest, widest, and longest points of the samples. Volume was calculated by multiplying height, width, and length of the samples. Cross-sections were calculated by multiplying the height and width of the samples.

### Protein loss

To quantify potential protein loss for the pocket clearing process, 10μL of the SDS clearing solution was collected from each sample every 3–4 days over the 6 weeks tissue clearing process. The amount of protein was determined for each timepoint and each tissue size using the Thermo Scientific Pierce BCA Protein Assay Kit (Thermo Scientific, Waltham, MA, USA). Next, the original protein content was determined by lysing the tissue using protein extraction buffer, Protease and Phosphatase Inhibitor. This protein content was also analyzed using a sample plate reader (FLUOstar Omega, BMG Labtech, Ortenberg, Germany) at a wavelength of 562 nm following protocol from the Thermo Scientific Pierce BCA Protein Assay Kit.

### Lightsheet fluorescence microscopy

For Lightsheet Fluorescence Microscopy (LSFM) imaging, the samples were removed from the SDS clearing solution and placed into PBST (PBS with Triton-X100), which was changed twice a day for 72 h to release any residual clearing solution. After this preparation, each sample was fixed at the end of a glass capillary at the epicardium side using super glue (Krazy Glue, Krazy Glue, USA). At the endocardium side, a metal bead was glued to weigh the sample down in order to prevent it from floating in the viscous, index-matching imaging solution (EasyIndex, LifeCanvas Technologies, Cambridge, MA, USA). The sample was then loaded vertically into the imaging chamber, with endocardium side facing down (Figure 1B).

A home-built light sheet microscope was used (28) for imaging. Briefly, a 488 nm illumination laser beam (OBIS 488 nm LS 60 mW, Coherent Inc., Santa Clara, CA, USA) was expanded and passed through a 50 mm cylindrical lens (ACY254-050-A, Thorlabs, Newton, NJ, USA) to generate a light sheet. The light sheet was projected into the sample using a 4x objective (CFI Plan Fluor 4x/0.13, Nikon, Melville, NY, USA). Then, autofluorescence signal from the sample was collected perpendicular to the light sheet by a 4x detection objective (CFI Plan Apo Lambda 4x/0.2, Nikon, Melville, NY, USA) and projected onto a sCMOS camera (Prime BSI, 6.5 μm pixel size, 2,048 × 2,048 pixel^2^ chip size, 95% quantum efficiency, Teledyne Photometrics, Tucson, AZ, USA) after passing through a green emission filter (ET 525/50, Chroma technology, Bellows Falls, VT, USA). Scattering images had better contrast then autofluorescence images and were used for analysis. To reduce striping artifacts, a resonant mirror (SC-30-10 × 10-20-1000, EOPC, Fresh Meadows, NY, USA) was used to pivot the light sheet at the sample plane (29). Images were also acquired using laser light scattered off the sample by removing the emission filter. All samples were scanned axially at 10 μm steps and tiled using a 4D stage (USB 4D stage, Picard industries, Albion, NY, USA).

## Image 3D reconstruction and helicity analysis

The imaged cross sections of the cleared samples were stitched in post processing utilizing Fiji (ImageJ) (30) using the “Grid/collection stitching” plugin (31) followed by resampling the 3D volume transmurally using the “Reslicing” function to generate width and height cross sections and further resized using the “Size” function for accurate representation of the cross sections from the endocardium to the epicardium of the tissue sample.

The transmurally resliced dataset was rotated to be orthogonal to the short axis plane before fiber orientation calculation to yield in-plane helix angle in the same axes (32). Fiber orientation was calculated using the OrientationJ (33) plugin for Fiji at 10 × 10 pixel blocks based on structure tensor analysis. Helix angle was derived from the dominant fiber orientation for every 10 × 10 block. The average helix angle of each slice was plotted at each transmural depth and the slope fitting this plot represented the helicity for each sample.

### Statistical analysis

Statistical analysis was performed using Prism Version 9. Statistical analysis of the percent change in volume, length, and cross-section were performed using the Mann-Whitney *U*-test (unpaired *t*-test).

Statistical analysis for protein loss was performed using Repeated Measures one-way ANOVA. The total protein loss was then analyzed using Multiple comparison 2-way ANOVA. The data was also analyzed using Multiple Mann-Whitney tests and was confirmed to be not statistically significant. Helical angle transmurality comparison analysis was performed using the Mann-Whitney *U*-test.

## Data availability statement

The raw data supporting the conclusions of this article will be made available by the authors, without undue reservation.

## Ethics statement

This animal study was reviewed and approved by the Ethical Review Board of Massachusetts General Hospital.

## Author contributions

JK, SP, and AS contributed equally to writing the manuscript. All authors helped in data collection and gave feedback regarding the manuscript.
