# Supplementary material for: Pocket CLARITY enables distortion-mitigated cardiac microstructural tissue characterization of large-scale specimens
Source: Front Cardiovasc Med. 2022 Nov 14;9:1037500. doi: 10.3389/fcvm.2022.1037500 (PMC9701703; doi:10.3389/fcvm.2022.1037500)
Supplement: Supplementary file 1 [file Data_Sheet_1.docx]

**SUPPLEMENTAL FIGURES**


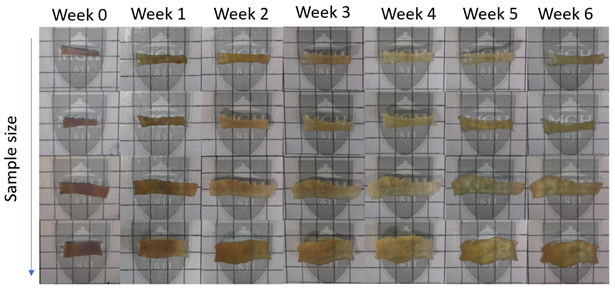


**Supplemental Figure 1. CLARITY of transmural left ventricle core from Week 0 to Week 6 of clearing.** CLARITY of transmural left ventricle core from Week 0 to Week 6 of clearing, each lattice indicating 5mm x 5mm. The epicardium is to the left and endocardium is to the right.


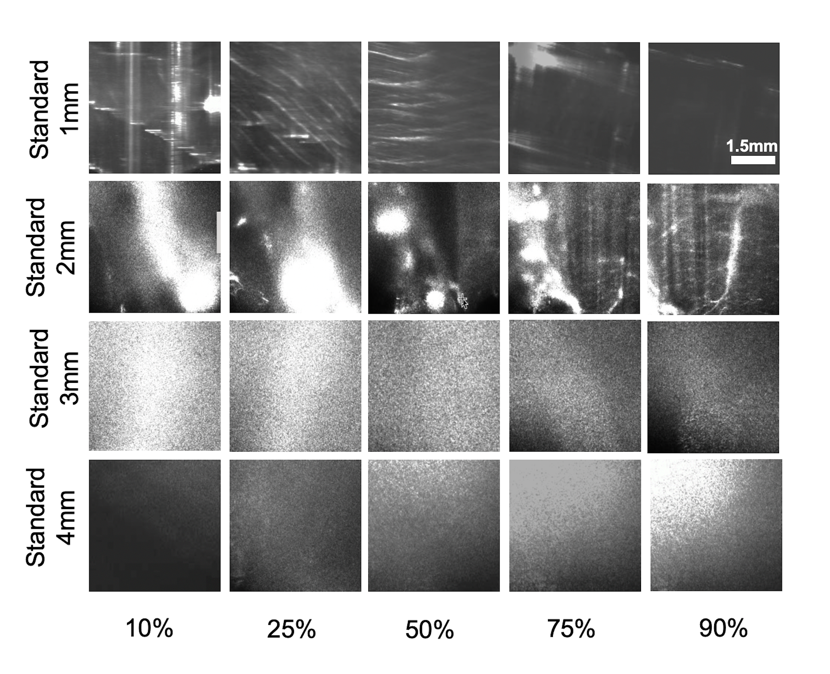


**Supplemental Figure 2. Transmural Optical Sections of CLARITY samples.** Longitudinal optical sections were resampled orthogonally into cross sections to reveal transmural optical sections of 1mm, 2mm, 3mm and 4mm CLARITY.


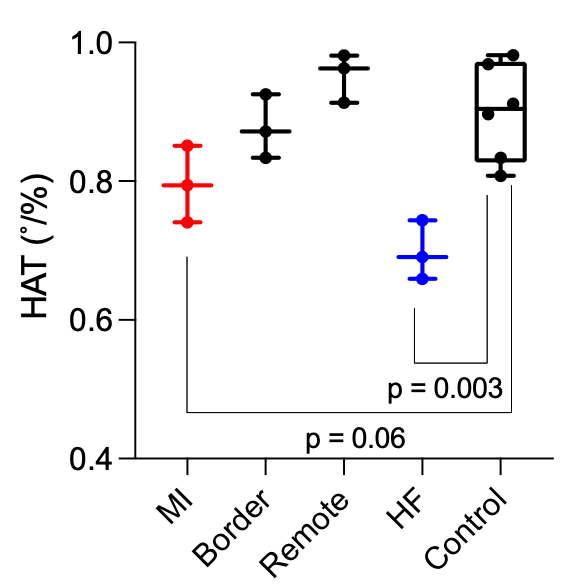


**Supplemental Figure 3. HAT Values for the MI, Border, Remote, HF, and Control samples.** The HAT values for the HF samples and the control were significant with a p-value of 0.003, while the HAT values for MI samples and control samples were not significant with p-value of 0.06.
